# Use of Large Language Models to Assess the Likelihood of Epidemics From the Content of Tweets: Infodemiology Study

**DOI:** 10.2196/49139

**Published:** 2024-03-01

**Authors:** Michael S Deiner, Natalie A Deiner, Vagelis Hristidis, Stephen D McLeod, Thuy Doan, Thomas M Lietman, Travis C Porco

**Affiliations:** 1 Department of Ophthalmology University of California, San Francisco San Francisco, CA United States; 2 Francis I. Proctor Foundation for Research in Ophthalmology University of California, San Francisco San Francisco, CA United States; 3 College of Letters and Science University of California, Santa Barbara Santa Barbara, CA United States; 4 Department of Computer Science and Engineering University of California, Riverside Riverside, CA United States; 5 American Academy of Ophthalmology San Francisco, CA United States; 6 Department of Epidemiology and Biostatistics University of California, San Francisco San Francisco, CA United States

**Keywords:** conjunctivitis, microblog, social media, generative large language model, Generative Pre-trained Transformers, GPT-3.5, GPT-4, epidemic detection, Twitter, X formerly known as Twitter, infectious eye disease

## Abstract

**Background:**

Previous work suggests that Google searches could be useful in identifying conjunctivitis epidemics. Content-based assessment of social media content may provide additional value in serving as early indicators of conjunctivitis and other systemic infectious diseases.

**Objective:**

We investigated whether large language models, specifically GPT-3.5 and GPT-4 (OpenAI), can provide probabilistic assessments of whether social media posts about conjunctivitis could indicate a regional outbreak.

**Methods:**

A total of 12,194 conjunctivitis-related tweets were obtained using a targeted Boolean search in multiple languages from India, Guam (United States), Martinique (France), the Philippines, American Samoa (United States), Fiji, Costa Rica, Haiti, and the Bahamas, covering the time frame from January 1, 2012, to March 13, 2023. By providing these tweets via prompts to GPT-3.5 and GPT-4, we obtained probabilistic assessments that were validated by 2 human raters. We then calculated Pearson correlations of these time series with tweet volume and the occurrence of known outbreaks in these 9 locations, with time series bootstrap used to compute CIs.

**Results:**

Probabilistic assessments derived from GPT-3.5 showed correlations of 0.60 (95% CI 0.47-0.70) and 0.53 (95% CI 0.40-0.65) with the 2 human raters, with higher results for GPT-4. The weekly averages of GPT-3.5 probabilities showed substantial correlations with weekly tweet volume for 44% (4/9) of the countries, with correlations ranging from 0.10 (95% CI 0.0-0.29) to 0.53 (95% CI 0.39-0.89), with larger correlations for GPT-4. More modest correlations were found for correlation with known epidemics, with substantial correlation only in American Samoa (0.40, 95% CI 0.16-0.81).

**Conclusions:**

These findings suggest that GPT prompting can efficiently assess the content of social media posts and indicate possible disease outbreaks to a degree of accuracy comparable to that of humans. Furthermore, we found that automated content analysis of tweets is related to tweet volume for conjunctivitis-related posts in some locations and to the occurrence of actual epidemics. Future work may improve the sensitivity and specificity of these methods for disease outbreak detection.

## Introduction

### Background

Conjunctivitis, while usually self-limiting, results in substantial societal costs [1,2] and can give rise to large outbreaks [2-7]. The detection of conjunctivitis epidemics can help reduce societal burden, prevent impacts on eye health, and act as a warning sign for emerging outbreaks of higher-risk systemic infectious diseases such as COVID-19. Recently, a study of a COVID-19 variant identified in 2023 that caused febrile illness and respiratory symptoms in children found conjunctivitis in 42.8% of the individuals who were affected [8,9].

The usual process of monitoring conjunctivitis outbreaks through individual case identification is costly; moreover, conjunctivitis is not, in general, a reportable disease in the United States (although gonococcal cause is reportable [10]). Low-cost digital approaches using public search and social media big data for surveillance could help fill this and other information gaps in eye health [11] by providing real-time information [11-13]. Previously, we found that an analysis of Google time series for relative search volume for conjunctivitis can identify outbreaks of conjunctivitis with differing ability based on the keywords, the country, and the size of the outbreak [14]; we also found that social media posts have been correlated with the clinical occurrence of conjunctivitis [15] and have reflected the seasonal occurrence of allergic and infectious conjunctivitis [16]. This suggested that a future system based on an analysis of web-based search frequency could be automated, reporting potential outbreaks worldwide.

By analyzing the content of social media posts during these detected candidate epidemics, we have observed that spikes in conjunctivitis-related search data can be caused by many factors besides outbreaks. These causes include media coverage, celebrity affliction, movie titles, artist names, and other factors not specific to infectious conjunctivitis. Any automated system aiming to detect, and alert about, potential epidemics based on search data would still require the monitoring of content during any suspected epidemic period to improve specificity. Previous research has suggested that web-based content can be useful in infectious disease surveillance [12,13,17-29]. Unfortunately, manual content analysis can be time-consuming, but available generative large language models (LLMs) could be assessed for their potential to assist with such a task in an automated fashion.

### The Aim of This Study

In this study, we investigated whether the analysis of geolocated time-series social media content [30] using LLMs could be used to accurately summarize the content of posts regarding conjunctivitis in general. To help refine our assessment of potential conjunctivitis outbreaks detected from search data in an automated fashion, we also investigated whether LLMs could assign a useful probability that a post’s content is specifically about a conjunctivitis outbreak [31]. We obtained tweets from 9 of the countries assessed in our previous study [14] and presented these to GPT-3.5 and GPT-4 (OpenAI) [32], which are transformers-based LLMs. We tested the hypotheses that automated content analysis using these models can yield a time series of elicited outbreak probabilities and that these probabilities are correlated with tweet frequency and the occurrence of known epidemics.

## Methods

### Data Collection

On the basis of our previous analysis [14], we chose 9 countries for which we knew the dates of conjunctivitis epidemics. We chose these to include both small countries and island territories as well as large countries; results for no other countries were analyzed. For these countries, we collected tweets from the Twitter microblogging service (subsequently rebranded as X) using the Brandwatch interface. To obtain posts about conjunctivitis, we used a Boolean query containing words in multiple languages representing conjunctivitis (eg, “conjuctivitis,” “conjuntivitis,” “conjuntivite,” and “pink eye”). We tailored this to exclude irrelevant content, such as that related to animals, and confounding content (such as celebrities having pink eye).

The full Boolean query is provided in Multimedia Appendix 1. Only tweets geolocated to each country were exported. The data cutoff window began on January 1, 2012 (January 1, 2013, for India), and ended on March 13, 2023. The data were exported on March 13, 2023, and the counts are summarized in Table 1. The corresponding epidemic start dates, presented in our previous study [14], are also included in Table 1.

**Table 1:** Summary of available tweets and known epidemics in the 9 study countries.

Location	Tweets, n	Epidemic start dates
India	4999	August 9, 2012; July 25, 2013; November 15, 2013; September 4, 2014; and April 9, 2017
Guam (United States)	282	May 15, 2014
Martinique (France)	336	May 14, 2017
Philippines	3976	August 27, 2015
American Samoa (United States)	68	April 1, 2014
Fiji	142	March 15, 2016
Costa Rica	1494	June 30, 2017
Haiti	512	May 15, 2017
Bahamas	385	May 15, 2017

### Data Analysis

#### Automated Content Analysis

We used the OpenAI LLMs GPT-3.5 (gpt-3.5-turbo-0301) and GPT-4 (gpt-4-0314), accessed through the application programming interface [33]. Another potentially comparable LLM, Google Bard, was not available through an application programming interface at the time we conducted our study. GPT-4 was available in limited beta release and was only used for prompt 1. We chose to use the less expensive GPT-3.5 as well as the newer, potentially more advanced GPT-4.

First, for each tweet, we directly elicited a probability that the tweet indicated a conjunctivitis outbreak. For this, we used prompt 1:

How certain are you that the single Tweet provided below is about a large multiperson outbreak of pink eye occurring at the time the tweet was posted? A single case with no other evidence of spread or other infected people should correspond to a somewhat low probability. Respond in the form of “Tweet: x%,” on a scale of 0% to 100%, and then provide a brief explanation of your answer. Given Tweet: <direct quote>

Second, we asked the model to simply assess the occurrence of an epidemic, based on the content of the tweet. This was prompt 2:

Answer if the tweet below is about a large multiperson outbreak of conjunctivitis, occurring at the time the tweet was made. A single case with no other evidence of spread or other infected people should correspond to a somewhat low probability. The response choices are: NO, not conjunctivitis outbreak (the tweet is irrelevant or indicates 0-1 cases of conjunctivitis max, not spreading or not occurring at the time the tweet was made); MAYBE conjunctivitis outbreak (uncertain, the tweet indicates maybe 2 or more cases of conjunctivitis, maybe spreading); YES conjunctivitis outbreak (the tweet indicates more than 1 case of conjunctivitis and/or spreading, and occurring at the time the tweet was made). For your answer, respond first with one of the three choices (NO, “not conjunctivitis outbreak,” MAYBE, “conjunctivitis outbreak,” YES, “conjunctivitis outbreak”) and then provide a brief explanation for your choice, including the type of disease if you say YES, “conjunctivitis outbreak.” Given Tweet: <direct quote>

Although the use of a continuous variable (elicited probability) from prompt 1 maximizes statistical power [34] compared with dichotomized data, we also included the results of the conceptually simpler prompt 2 along with the results of prompt 1 for comparison.

For both prompts 1 and 2, we replaced *<direct quote>* with each of the 12,194 tweets in turn, collecting all responses. For all queries, we used a *top_p* of 0.9 (the default value) and a temperature of 0.

To provide illustrative examples, we divided the tweets into groups with GPT-3.5–derived percentages of 0%, between 0% and 70% (exclusive), and >70% and randomly selected 3 tweets from each group. We removed specific identifying information from each tweet and lightly edited them to reduce discoverability [35]; we note that these tweets were public. Samples of these redacted tweets and LLM responses to prompt 1 for them were prepared solely for the illustration of LLM replies to the 2 prompts. Only replies to the original unredacted tweets were used in all analyses presented in this study.

#### Human Rater Validation of GPT Classification and Scoring

To validate the resulting conjunctivitis epidemic probabilities and classifications by GPT-3.5 and GPT-4 of the tweets, 2 human raters participated in a modified Delphi session. During the session, the raters manually reviewed a random sample of tweets, classified them into the same categories as the GPT models (“NO,” “YES,” and “MAYBE” conjunctivitis outbreak), and assigned a conjunctivitis epidemic probability score (0%-100%) to each. The human and GPT categorizations and scores were then compared.

We asked the 2 human raters to independently read each tweet, using the same prompts that were provided to the LLMs. For the testing set used, a random selection of tweets was stratified by country and by the elicited probabilities from GPT-3.5 to ensure that as close to a maximum of 7 tweets that scored >50% and 7 tweets that scored <50% were included from each country (126 tweets in total). The sample size was chosen to provide a CI half-width of approximately 0.05 for estimated proportions near 0.5. Similarly, separate training sets of independent tweets were generated (18 per set). Only English-language tweets were used in validation. Training and testing sets were used as described in the following paragraph.

The raters first trained together on the first training set, assigning classification and probability scores via a Qualtrics survey (Qualtrics International Inc). A facilitated group discussion for the raters then followed, to reconcile disagreements in the categorization and scores as well as to gain familiarity with the discussion on Twitter (ie, to become aware of the language and components, such as hashtags and sarcasm, used in these posts). The raters subsequently completed a second iteration of the training with the second training set, followed by a similar brief discussion as before so that a general agreement was reached. We then provided the testing set in a separate Qualtrics survey (excluding any tweets used in training the raters) to the raters. Each rater assigned classification and probability scores to each post in the testing set, masked to the results of other raters and that of the machine and without any discussion.

#### Statistical Analysis

In time-series data of tweet volume about a disease, we expect an increase in the weekly count of posts about the disease during an epidemic compared with nonepidemic periods [36]. Therefore, as an assessment of the ability of the GPT models to assign higher probabilities to tweets in weeks where there may be more likely to be an epidemic (higher counts of total tweets per week) as well as to assign lower probabilities to weeks less likely to have an epidemic (low total counts of tweets per week), we asked whether the weekly count of posts about pink eye correlated with the scores assigned to that week by the LLMs. To calculate weekly values from the elicited probabilities from each of the GPT models, we first removed highly repetitive tweets as follows: we removed usernames beginning with @ from the content and then removed all tweets with duplicated content. From the remaining tweets, we averaged all those values for each week. Weeks with no tweets at all were scored as having a mean of 0. Elicited percentages were treated as continuous variables in statistical analyses [34]. We converted prompt 2 results to numerical values (to allow for correlation analysis) by assigning values: 0=“NO,” 1=“MAYBE,” and 2=“YES.”

For each country in Table 1, we constructed an indicator variable, which was 1 for any week an epidemic was believed to have started and for 3 weeks after. We then calculated the Pearson correlation between the number of tweets per week and the mean LLM-derived conjunctivitis outbreak probability score (prompt 1) or outbreak classification (prompt 2) for each week. We used time-series bootstrap [37] with a width of 4 to calculate CIs. We also correlated the weekly epidemic indicator variable with the mean elicited percentages obtained from GPT-3.5 and GPT-4 in response to prompt 1; we note that because we have no complete listing of all epidemics, the correlation will unavoidably underestimate the true relation between the occurrence of an epidemic and any content analysis measure. In addition, we included the volume of tweets matching our query as an outcome variable when comparing with epidemic indicator variables. As a sensitivity analysis, we conducted analysis without removing duplicates and without considering weeks with 0 tweets as having a mean of 0. Estimated Pearson correlations for the time series were compared between GPT-3.5 and GPT-4 by subtracting the estimates for each country and conducting the Wilcoxon signed rank test.

### Ethical Considerations

The work carried out for this study was conducted after receiving approval from the University of California San Francisco Institutional Review Board (14-14743).

## Results

### Descriptive Summary of GPT Model Responses

The overall ability of the 2 LLMs to respond in the format that we desired was high. For prompt 1, GPT-3.5 provided usable responses 99.66% (12,153/12,194) of the time, whereas GPT-4 provided usable responses 100% (12,194/12,194) of the time. For prompt 2, GPT-3.5 provided usable responses 98.67% (12,032/12,194) of the time. We observed that the ability to summarize the content of the tweets seemed quite reliable and surprisingly nuanced; selected examples of prompt 1 responses, based on the redacted tweets described previously, are provided in Table 2. For comparison, prompt 2 responses to these same tweets are provided in Table S1 in Multimedia Appendix 1.

**Table 2:** Selected actual GPT-3.5 and GPT-4 responses to prompt 1 for typical tweets from our data set (for this table, actual usernames, locations, and other identifiers were modified to avoid presenting identifying information).

Tweet	GPT-3.5 response	GPT-4 response
All undergrads under me got conjunctivitis. So not allowing to theatre and class. Some who r nt effected also act like they got. Managing class all alone. Im jealous seeing them enjoying.	Tweet: 70% Explanation: The tweet mentions that “All undergrads under me got conjunctivitis” which suggests a large multiperson outbreak. Additionally, the author mentions not allowing them to attend class or theater, which implies that the outbreak is significant enough to warrant such measures. However, there is no mention of any official reports or confirmation of the outbreak, so there is still some uncertainty.	Tweet: 60% Explanation: The tweet suggests that multiple undergraduates have contracted conjunctivitis (pink eye), which indicates a possible outbreak. However, the tweet also mentions that some students are pretending to have the condition, which makes it difficult to determine the true extent of the outbreak. Additionally, this is just one tweet and not enough to confirm a large multiperson outbreak.
Pharmacies ran out of eye drops for conjunctivitis as those infected continue to go to work, school, gatherings, and so on. They affect others–urgh!	Tweet: 80% Explanation: The tweet suggests that there is a shortage of eye drops for conjunctivitis, which implies that there is a high demand for them. The fact that those infected continue to go to work, school, and gatherings suggests that there is a high likelihood of transmission to others, leading to a large multiperson outbreak. However, without additional information, it is difficult to determine the exact size of the outbreak, hence the probability of 80%.	Tweet: 80% Explanation: The tweet suggests a higher probability of a large multiperson outbreak of pink eye, as it mentions pharmacies running out of eye drops for conjunctivitis and infected individuals continuing to attend work, school, and gatherings, which could lead to the spread of the infection. However, without further information or confirmation, there is still some uncertainty.
@user conjunctivitis??	Tweet: 50% Explanation: The tweet alone does not provide enough information to determine the certainty of a large multiperson outbreak of pink eye. The tweet only mentions one case of conjunctivitis and does not provide any additional information about the spread or other infected people. Therefore, there is a moderate probability of a large multiperson outbreak of pink eye occurring at the time the tweet was posted.	Tweet: 10% Explanation: The tweet only mentions “conjunctivitis” with a question mark, which provides very little information about the context or any potential outbreak. There is no mention of multiple people being affected or any indication of a large-scale event. Therefore, the certainty of this tweet being about a large multiperson outbreak of pink eye is quite low.
１日家にいたーひさびさのひきこもりやっぱ結膜炎気になって つきっきりしてたー だいぶ寝たー	Tweet: 10% Explanation: The tweet is in Japanese, but based on the use of the word “結膜炎” (conjunctivitis or pink eye) and the fact that the person stayed at home all day and rested, it is possible that they had a case of pink eye. However, there is no mention of other people being infected or any indication that this is a large multiperson outbreak. Therefore, the probability of this tweet being about a large multiperson outbreak of pink eye is relatively low at 10%.	Tweet: 10% Explanation: The tweet is in Japanese and translates to “I stayed home all day. It’s been a while since I’ve been a shut-in. I was worried about conjunctivitis (pink eye) and stayed close. I slept a lot.” This tweet only mentions a single case of pink eye and does not provide any information about a large multiperson outbreak. Therefore, the probability is low at 10%.
do i have pink eye or could it be my allergies again?	Tweet: 0% Explanation: The given tweet does not provide any evidence of a large multiperson outbreak of pink eye. The tweet only mentions a personal experience of the individual and does not suggest any spread or other infected people. Therefore, the probability of the tweet being about a large multiperson outbreak of pink eye is very low, at 0%.	Tweet: 5% Explanation: The tweet only mentions the possibility of the user having pink eye or allergies, but there is no mention of a large multiperson outbreak or any other infected people. Therefore, the probability is very low.
Did you know that the first case of viral conjunctivitis was found in 1902? It is 2020 and “science” still has no “cure” for it!	Tweet: 0% Explanation: The given tweet does not provide any evidence or mention of a large multiperson outbreak of pink eye occurring at the time the tweet was posted. It only talks about the history of viral conjunctivitis and the lack of a cure for it. Therefore, the probability of the tweet being about a large multiperson outbreak of pink eye is very low, at 0%.	Tweet: 0% Explanation: The tweet provided does not mention any current outbreak of pink eye or any indication of multiple infected people. It only discusses the first case of viral conjunctivitis found in 1902 and the lack of a cure in 2020.

### Human Rater Validations of GPT-Assigned Scores and Classifications That a Tweet Was About Conjunctivitis Outbreaks

We found that, overall, the LLMs were less likely than the human raters to assign the extreme values of 0% or 100%. Moreover, GPT-3.5 chose larger values than GPT-4 for the validation sample. A descriptive summary of the validation sample is presented in Table 3.

Next, we computed correlations between the elicited percentages of the 2 human raters and the responses of GPT-3.5 and GPT-4 to prompt 1. Overall, the percentages derived from the replies of both GPT-3.5 and GPT-4 had a correlation coefficient of at least 0.6 with those of the human raters, although the responses of GPT-4 were more correlated with those of the human raters than those of GPT-3.5. The correlation of the percentages of GPT-4 were roughly as correlated with those of the human raters as the results of the human raters were with each other. These validation set results are summarized in Table 4. As a measure of interrater reliability for prompt 2, the estimated unweighted Cohen κ value was 0.64 (*P*<.001) for a comparison of the 2 human raters. The Cohen κ value for a comparison of the results of rater 1 with those of GPT-3.5 for prompt 2 was 0.51 (*P*<.001), and for a comparison of the results of rater 2 with those of GPT-3.5 for prompt 2, the Cohen κ value was 0.48 (*P*<.001).

**Table 3:** Validation with human raters: summary of grading. The proportion of the total corpus of validation testing set tweets (n=126) assigned by human raters and the GPT models to 0% and 100% probability that a tweet is about an outbreak is shown, along with the median (IQR) percentage assigned.

Measurement	Rater 1	Rater 2	GPT-3.5, prompt 1	GPT-4, prompt 1
Ratings of 0%, n (%)	4 (0.3)	5 (0.4)	1 (0.1)	0 (0)
Rating (%), median (IQR)	30 (0-90)	10 (0-100)	55 (10-70)	10 (10-30)
Ratings of 100%, n (%)	3 (0.2)	4 (0.3)	0 (0)	0 (0)

**Table 4:** Validation with human raters: correlation (Pearson r). Correlation matrix of elicited percentages from human raters and GPT models using prompt 1, based on the validation set (n=126).

Variable	Human 1	Human 2	GPT-3.5, prompt 1	GPT-4, prompt 1
Human 1, *r* (95% CI)	1	0.77 (0.68-0.83)	0.60 (0.47-0.70)	0.73 (0.64-0.80)
Human 2, *r* (95% CI)	0.77 (0.68-0.83)	1	0.53 (0.40-0.65)	0.77 (0.68-0.83)
GPT-3.5, prompt 1, *r* (95% CI)	0.60 (0.47-0.70)	0.53 (0.40-0.65)	1	0.77 (0.68-0.83)
GPT-4, prompt 1, *r* (95% CI)	0.73 (0.64-0.80)	0.77 (0.68-0.83)	0.77 (0.68-0.83)	1

### Descriptive Summaries of GPT-3.5 and GPT-4 Probabilities and Classifications

**Figure 1:**
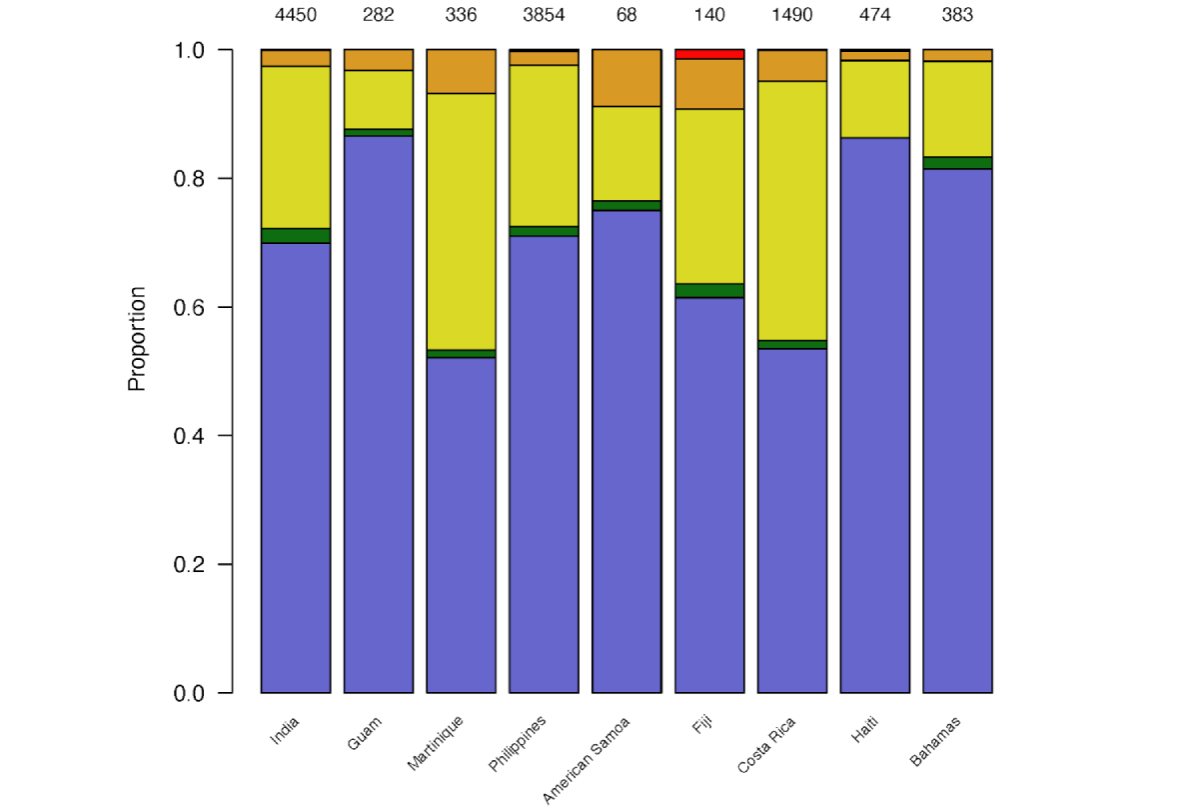
Distributions of elicited epidemic probability assigned to tweets by GPT-3.5 for each country (12,194 tweets in total) using prompt 1. Light blue: 0% to 20%, green: 21% to 40%, yellow: 41% to 60%, orange: 61% to 80%, and red: 81% to 100%. The total count is placed at the top of each bar.

**Figure 2:**
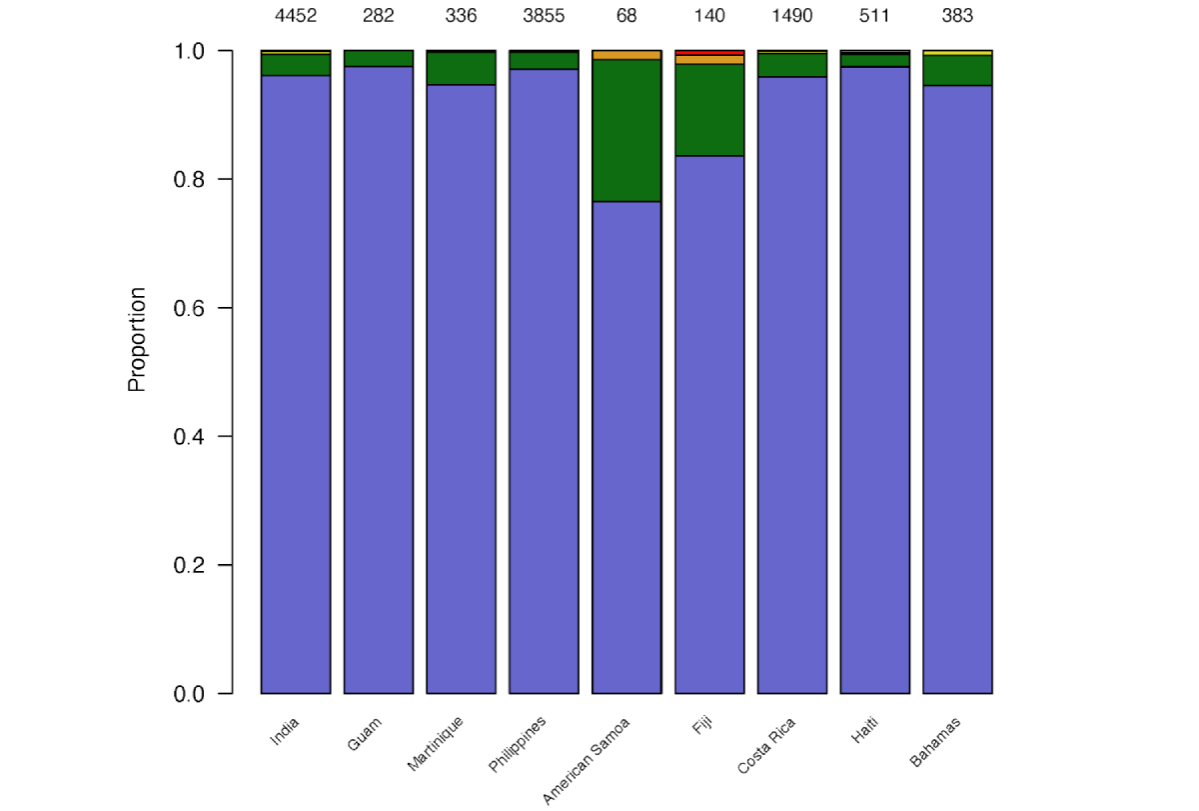
Distributions of elicited epidemic probability assigned to tweets by GPT-4 for each country (12,194 tweets in total) using prompt 1. Light blue: 0% to 20%, green: 21% to 40%, yellow: 41% to 60%, orange: 61% to 80%, and red: 81% to 100%. The total count is placed at the top of each bar.

For each of the 9 countries, summaries of the elicited percentages for the full set of tweets using GPT-3.5 and GPT-4 are shown in Figure 1 and Figure 2, respectively. The models provided low percentages (≤20%) for most of the tweets (7922/12194, 65.0% for GPT-3.5; 11070/12194, 90.8% for GPT-4) in all countries. Of the 12,194 tweets, 677 (5.55%) were removed because they were highly repetitive. From the remaining 11,517 tweets, the overall mean percentage elicited was 21%, with a median percentage of 10% (IQR: 5-50%). For prompt 1, neither GPT-3.5 nor GPT-4 provided any elicited percentages of 100%. Both showed profound final digit preference; in only 1 case did GPT-3.5 provide a percentage that did not end in 0 or 5, and all from GPT-4 ended in 0 or 5.

**Figure 3:**
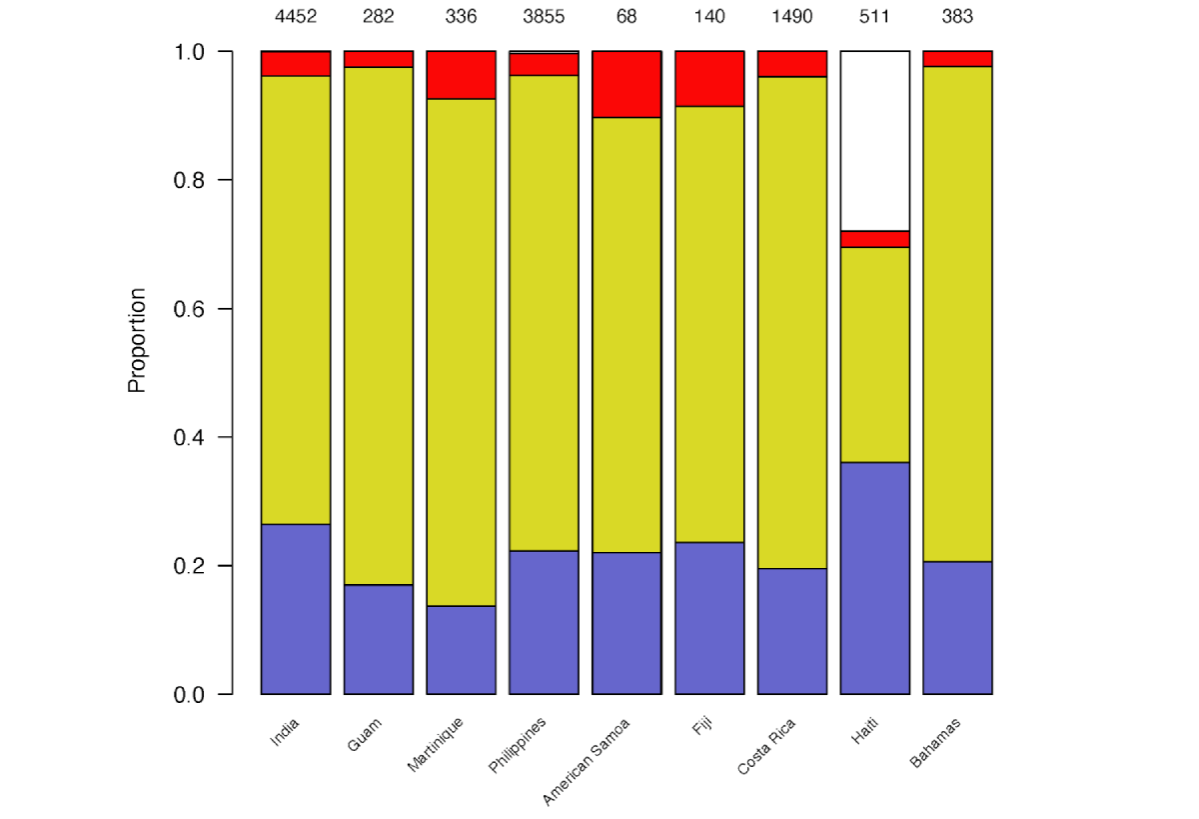
Distributions of elicited epidemic classifications assigned to tweets for GPT-3.5 for each country (12,194 tweets in total) using prompt 2. Light blue: "NO," yellow: "MAYBE," red: "YES," white: "missing." The total count is placed at the top of each bar.

In response to prompt 2, where we simply asked the LLM to classify each tweet as “YES,” “NO,” or “MAYBE” regarding an outbreak of conjunctivitis, the distribution of classifications assigned to each tweet by GPT-3.5 is shown in Figure 3. Of note, in 162 (1.41%) of the 11,517 tweets, the LLM’s response did not begin with 1 of the 3 requested words, and we treated these as missing (although in all cases, the LLM responded with an explanation of why it was difficult to be sure of the answer and therefore did not choose 1 of the 3 response options).

### Correlation of Results for Tweets Between Models and Between Prompt 1 and Prompt 2

At the level of individual tweets, the probabilities assigned by GPT-3.5 and GPT-4 based on prompt 1 were highly correlated, with a Pearson *r* value of 0.42 (95% CI 0.41-0.44). To compare the results elicited from GPT-3.5 for prompts 1 and 2 per tweet, we converted the elicitations from prompt 2 to numerical values. Specifically, we assigned the following values: 0=“NO,” 1=“MAYBE, and 2=“YES.” We found a correlation of 0.45 (95% CI 0.43-0.46) between the prompt 1–elicited probabilities and the prompt 2–elicited classifications.

### Comparisons of Elicited Epidemic Probability and Epidemic Classification Results per Tweet Between Models and Between Prompt 1 and Prompt 2

We next compared the elicited epidemic probabilities from the LLMs with weekly tweet volume based on our search, as described in the *Methods* section. We computed the Pearson correlation of the number of tweets meeting the search criteria, as well as the mean elicited percentages for GPT-3.5 and GPT-4 in response to prompt 1. We also used a binary indicator of whether GPT-3.5 responded “YES” to prompt 2. The estimated correlations for GPT-3.5 using prompt 1 ranged from 0.10 (India) to 0.53 (American Samoa [United States]); for GPT-4 using prompt 1, the estimated correlations ranged from 0.18 (India) to 0.64 (Guam [United States]), with broadly higher correlations seen in GPT-4 (*P*=.004, Wilcoxon signed rank test). The results for each of the 9 countries are shown in Table 5. When weeks containing 0 tweets were excluded, the results were similar (refer to Table S2 in Multimedia Appendix 1). Similarly, when we did not exclude duplicated or highly repetitive tweets, the results were similar (although slightly lower; results not shown).

**Table 5:** Correlation of weekly tweet volume and elicited outbreak percentages by the GPT models (refer to the text for details).

Country	GPT-3.5, prompt 1, weekly mean, *r* (95% CI)	GPT-4, prompt 1, weekly mean, *r* (95% CI)	GPT-3.5 “YES,” prompt 2, weekly mean, *r* (95% CI)
India	0.10 (−0.00 to 0.29)	0.18 (0.09 to 0.37)	0.04 (−0.01 to 0.13)
Guam (United States)	0.42 (0.34 to 0.57)	0.64 (0.55 to 0.79)	0.08 (0.04 to 0.18)
Martinique (France)	0.36 (0.31 to 0.66)	0.45 (0.40 to 0.81)	0.13 (0.08 to 0.26)
Philippines	0.14 (0.07 to 0.21)	0.23 (0.13 to 0.32)	0.05 (−0.02 to 0.13)
American Samoa (United States)	0.53 (0.39 to 0.89)	0.60 (0.52 to 0.94)	0.20 (0.09 to 0.85)
Fiji	0.33 (0.30 to 0.67)	0.42 (0.37 to 0.81)	0.18 (0.13 to 0.59)
Costa Rica	0.17 (0.13 to 0.33)	0.22 (0.16 to 0.43)	0.08 (0.04 to 0.16)
Haiti	0.12 (0.08 to 0.37)	0.29 (0.24 to 0.66)	0.05 (0.03 to 0.12)
Bahamas	0.41 (0.36 to 0.50)	0.58 (0.52 to 0.71)	0.06 (0.03 to 0.11)

### Comparisons of Elicited Epidemic Probabilities With Known Epidemics

We next calculated the Pearson correlation of the weekly indicator variable with the mean elicited percentage for GPT-3.5 and GPT-4 in response to prompt 1. We note that because conjunctivitis is not typically reportable (except under special circumstances), no comprehensive set of known epidemics is available—weeks coded as *not epidemic related* could have contained epidemics. As conjunctivitis outbreaks no longer seem to be reported on the Program for Monitoring Emerging Diseases (ProMED) system, we restricted the analysis to the same time period as our earlier report [14]. The correlations with these epidemic indicators were smaller than those with the tweet counts and were effectively 0 in India, Costa Rica, Martinique (France), and the Philippines; the correlations were substantial for American Samoa. Smaller but nonetheless indicative results were found for Fiji, Guam, and Haiti (for GPT-4). For large nations, we found correlations that were lower than those for small countries or island territories, as expected based on our earlier findings [14]. As before, broadly higher correlations were found for GPT-4 (*P*=.004, Wilcoxon signed rank test). A summary of these correlations is presented in Table 6.

In Table 7, for each country, we computed the average of available elicited probabilities for the months containing a known epidemic start date and for the months without. For 8 (89%) of the 9 countries, this average was larger in the months with an epidemic start date than in the months without. To potentially improve specificity, we also calculated the mean of only those elicited probabilities that were ≥51% (in an unprespecified analysis). These findings are shown in the last 2 columns of Table 7; of the 9 countries, 5 (56%) had a much higher difference between the epidemic and nonepidemic months.

**Figure 4:**
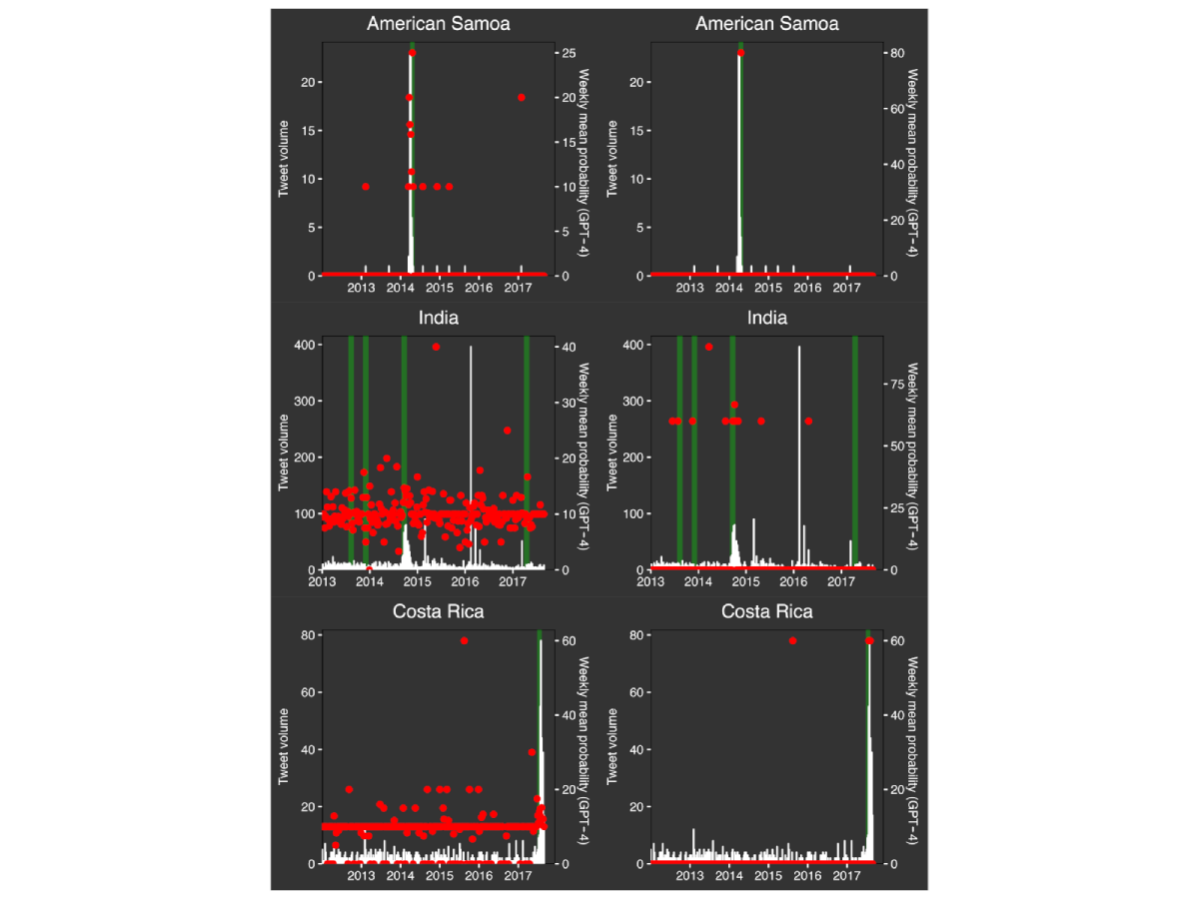
Representative time series plots of average weekly elicited likelihood scores for GPT-4 for 3 countries (American Samoa, India, and Costa Rica) using either all output results (column 1) or only values >50% (column 2). Red points: weekly average elicited probability for any week with at least 1 post for all values (column 1) or for only values >50% (column 2), white bars: number of tweets per week, and green band: reported epidemic period.

Figure 4 shows weekly mean elicited probabilities compared with epidemic and nonepidemic weeks and with weekly tweet volume for 3 selected countries. In the left column, we show in red the weekly mean of all available GPT-4–derived percentage likelihoods (with 0 when there are none); in the right column, we show the mean of all GPT-4 percentage likelihoods that are ≥51%. The green bands indicate epidemic periods (4 weeks before through 6 weeks after the reported start date of known epidemics). Not all conjunctivitis outbreaks are known and reported. For American Samoa, high weekly likelihood values corresponded with the peak in tweet volume and the known outbreak, whereas for some larger countries, such as India, this was not as apparent. In general, plots of weekly means of all likelihoods >51% provide a potentially more useful visualization of likely epidemics.

**Table 6:** Correlation of weekly mean GPT-3.5– and GPT-4–elicited epidemic probabilities with a weekly epidemic indicator (a time series taking the value 1 for the first 4 weeks of known reported outbreaks and 0 otherwise).

Country	GPT-3.5, prompt 1, weekly mean, *r* (95% CI)	GPT-4, prompt 1, weekly mean, *r* (95% CI)	GPT-3.5 “YES,” prompt 2, weekly mean, *r* (95% CI)
India	−0.03 (−0.13 to 0.08)	−0.00 (−0.16 to 0.12)	−0.00 (−0.05 to 0.07)
Guam (United States)	0.05 (0.01 to 0.10)	0.13 (0.07 to 0.22)	−0.01 (−0.02 to −0.00)
Martinique (France)	0.03 (−0.02 to 0.09)	0.05 (−0.02 to 0.11)	0.02 (−0.01 to 0.07)
Philippines	0.06 (0.01 to 0.10)	0.07 (0.02 to 0.13)	0.01 (0.00 to 0.03)
American Samoa (United States)	0.40 (0.16 to 0.81)	0.60 (0.29 to 0.85)	0.20 (−0.00 to 0.94)
Fiji	0.13 (−0.01 to 0.26)	0.24 (0.08 to 0.42)	0.08 (−0.01 to 0.57)
Costa Rica	0.05 (0.03 to 0.10)	0.06 (0.03 to 0.12)	0.07 (0.04 to 0.15)
Haiti	0.01 (−0.02 to 0.07)	0.09 (0.03 to 0.16)	−0.01 (−0.02 to −0.00)
Bahamas	0.04 (0.01 to 0.09)	0.07 (0.01 to 0.14)	0.01 (−0.01 to 0.04)

**Table 7:** Average monthly mean values for GPT-3.5 and GPT-4 of all elicited epidemic probabilities for months not containing a known epidemic start date (column 2) compared with months with a known epidemic start date (column 3). Monthly means of only those elicited probabilities that were ≥51% are shown for months not containing a known epidemic start date (column 4) compared with months with a known epidemic start date (column 5).

Country	Monthly mean (SD) of all probabilities, nonepidemic months, GPT-3.5	Monthly mean (SD) of all probabilities, epidemic months, GPT-4	Monthly mean (SD) of probabilities >51%, nonepidemic months, GPT-3.5	Monthly mean (SD) of probabilities >51%, epidemic months, GPT-4
India	8.4 (4.1)	9.3 (5.3)	7.2 (20.8)	36 (32.8)
Guam (United States)	7.6 (4.4)	13.6 (N/A^a^)	0 (0)	0 (N/A)
Martinique (France)	6.2 (5.2)	10 (N/A)	0.9 (7.3)	0 (N/A)
Philippines	9.8 (1.1)	13.7 (N/A)	8.2 (21.0)	80 (N/A)
American Samoa (United States)	1.1 (3.8)	16.4 (N/A)	0 (0)	80 (N/A)
Fiji	4.1 (6.5)	19.1 (N/A)	0 (0)	75 (N/A)
Costa Rica	10.5 (1.4)	11.9 (N/A)	2.7 (12.5)	0 (N/A)
Haiti	7.1 (4.9)	9.5 (N/A)	1.1 (9.1)	60 (N/A)
Bahamas	8.2 (4.3)	5 (N/A)	0.9 (7.3)	0 (N/A)

^a^N/A: not applicable.

## Discussion

### Principal Findings

Our main findings, with regard to the objectives and hypotheses stated in the *Introduction* section, are as follows.

1. We found that LLMs can be used to assess Twitter content related to conjunctivitis in general and in relation to infectious outbreaks of conjunctivitis. We found that we could elicit percentages representing the probability of an outbreak on a regional basis (in the sense of quantifying an uncertain judgment).
2. The 2 LLMs we examined (GPT-3.5 and GPT-4) showed substantial correlation with each other’s assessments of the likelihood of a conjunctivitis outbreak, as well as with the assessments of the 2 human raters.
3. We also found that these correlated with the results of other conjunctivitis-related prompts. In addition, we found evidence that the mean elicited percentages positively correlated with conjunctivitis-related tweet volume.
4. We also found evidence that these percentages correlate with known epidemics, particularly in selected small countries or island territories.

Our results suggest that our approach using a generative LLM (GPT-3.5 or GPT-4) could be used to both thematically define the contents of eye health–related tweets and assign Bayesian probability scores and classifications to help identify if a tweet is mentioning an eye disease outbreak. In view of the better performance of GPT-4 in benchmarks and tests [38-41], it is reassuring that the results from GPT-4 yielded higher correlations with tweet volume than GPT-3.5 with the same prompt. This study adds to a growing literature regarding the use of LLMs for analysis of social media posts related to health [42-45] (in our case, the assigning of a measure of health risk in addition to the interpretation of content). Future studies could explore the potential of the use of LLMs to assess the weekly content of posts about infectious eye cases to score the probability of an outbreak on a regional basis or as a low-cost weekly surveillance approach to help detect ocular epidemics. This could also validate suspected ocular epidemics determined from other web-based data sources [14]. This approach could also be applicable, in concert with topic modeling, to thematically define the content of posts regarding eye health risk. Such methods could allow for scalable thematic assessment of large sets of posts (eg, inductive content analysis [46] beyond the scope practical for time-consuming human analysis) to characterize current and emerging eye health topics of interest to the public with specific eye conditions. Topics could be scored for factors such as toxicity in an unbiased fashion.

Future studies should assess the ability of our model to use other sources of data (web-based discussion groups, forums, or blogs) to interpret and assess the likelihood of eye disease outbreaks or other emerging eye health risks. In addition, we could explore the ability of these models to classify other key informative features of an outbreak, such as health severity, etiology, or size. Although we have chosen conjunctivitis as a model (and certainly conjunctivitis outbreaks can act as a harbinger of a systemic and higher-risk disease), the principles used to develop this model can be applied to identify outbreaks of symptoms associated with a wide range of localized or systemic diseases that pose severe population health risk or threaten a pandemic [8,9,47], especially when these symptoms may be nonreportable.

This study highlights a relatively new use of LLMs for infodemiology and suggests potential for more efficient assessment of social media than in prior works; for example, scalable thematic assessment of large sets of posts could be completed by LLMs with less manual effort required than in prior studies [46]. As LLMs continue to be developed, we anticipate that the quality of such assessments by LLMs will continue to improve and that costs will fall. In addition, new discoveries about improving methods of prompting LLMs for better results are steadily emerging. Investments in automated content screening of microblog posts, as well as other public social media, blog, and forum data, may be warranted as an additional channel of potentially useful information for disease outbreak surveillance. Such methods could be particularly useful for other nonreportable conditions.

### Limitations

Our findings are subject to several limitations beyond those inherent in the selection of our 9 countries. Some relevant tweets may have been omitted because of our efforts to remove cinematic, celebrity-related, and other irrelevant content, and we note that an important potential application of LLMs is to help identify such content for elimination. It is also possible that our original query was missing some conjunctivitis-related keywords for some of the languages used in the countries included in our study, leading us to obtain low counts of posts about conjunctivitis in some languages. Future studies could further explore and expand keywords in other languages to improve our data signal for use in LLM analyses. Our prompts could be further optimized for the elicitation of probability scores from the LLMs with improved results [48]. Another limitation we found was that the LLM-elicited percentages did not correlate as well with known epidemics in large countries as they did with known epidemics in selected small countries or island territories. A possible reason for this could be that small disease outbreaks in large countries may occur frequently but go undetected when analyzing content for the entire country—this suggests that analysis of posts geolocated to smaller regions may prove more useful for detecting disease outbreaks in large countries.

In addition, tweets from some of the countries (eg, India, Martinique, Haiti, and Costa Rica) contained substantial content in other languages, and the current generations of the GPT models are somewhat less skillful in non-English languages. We note, however, that the models were entirely capable of translating and explaining content in many languages, which included Japanese, Marathi, and others in our sample [49,50], although we note a higher fraction of unusable replies for Haiti for GPT-3.5, prompt 2. Additional sources of social media data beyond Twitter could improve coverage and sensitivity. We also note that although the current LLMs were capable of replying with probabilities (expressed as percentages) seemingly indicating a degree of belief—with such values correlated with those of human raters—we have no evidence that these probabilities are calibrated (in the sense that the empirical relative frequency of true epidemics among tweets classified as probability *X* is, in fact, *X*). Finally, no complete database exists for known conjunctivitis outbreaks; therefore, it is not possible to precisely evaluate the sensitivity or specificity of our methods at this time.

### Conclusions

Our findings suggest that GPT prompting can efficiently assess the content of social media posts and possible disease outbreaks to a degree of accuracy comparable to that of humans. Furthermore, we found that the results of our automated content analysis of tweet content is related to tweet volume for conjunctivitis-related posts in some locations as well as to the occurrence of actual epidemics. Future work may improve the sensitivity and specificity of these methods. The approaches presented in this manuscript suggest the potential to leverage LLMs to assess social media or forum posts not only for automated and highly efficient identification of infectious eye disease outbreaks and other emerging eye health risks but also to detect outbreaks of high-risk diseases or classify key epidemiological characteristics of cases during outbreaks. This could improve timely identification of the most severe disease outbreaks, enabling localized action for mitigating impact on human health.

## Appendix

Multimedia Appendix 1 Boolean query and additional tables.

## Abbreviations

API: application programming interface
GPT: Generative Pre-trained Transformers
LLM: large language model
ProMED: Program for Monitoring Emerging Diseases

## Data Availability

The primary social media data sets analyzed during this study are not publicly available owing to our terms of use agreement with the Brandwatch platform but are available from the corresponding author using a data sharing agreement on reasonable request. We have placed the posts in the Qualitative Data Repository via the University of California San Francisco.
