# Supplementary material for: Use of Large Language Models to Assess the Likelihood of Epidemics From the Content of Tweets: Infodemiology Study
Source: J Med Internet Res. 2024 Mar 1;26:e49139. doi: 10.2196/49139 (PMC10943433; doi:10.2196/49139)
Supplement: Multimedia Appendix 1 [file jmir_v26i1e49139_app1.pdf]

Appendix

Boolean query

The following was our Boolean query:

("aankh aana" OR "azoumounou" OR "Bindehautentzündung" OR "bindhinneinflammation" OR "bindvliesontsteking" OR "congiuntivite" OR "conjonctivite" OR "conjunctivită" OR "conjunctivitis" OR "conjuntivite" OR "conjuntivitis" OR "je woz" OR "konjonktivit" OR "konjuktiviti" OR "konjuktivitis" OR "konjunktivitis" OR "konjunktivīts" OR "konjunktivitt" OR "konjunktyvitas" OR "kötőhártya-gyulladás" OR "œil rose" OR "pinkeye" OR "pink eye" OR "sidekalvontulehdus" OR "sidekestapõletik" OR "Viêm kết mạc" OR "viem ket mac" OR "zánětspojivek" OR "zapaleniespojówek" OR "zápalspojiviek" OR "конјуктивит " OR "конъюнктивит " OR "конюнктивит " OR "التهابالملتحمة" OR "آنکھکالالہوجانا" OR " आखका ला लहो जा न" OR " ਅੱਖਦਾ ਲਾ ਲਹੋਜਾ ਣਾ" OR "ตาแดง " OR "결막염 " OR "結膜炎 " OR "結膜炎 " OR "结膜炎 ") AND -RT AND -$ AND -http AND -dog AND -dogs AND -cat AND -cats AND -sheep AND -cow AND -cows AND -pig AND -pigs AND -livestock AND -goat AND -goats AND -bull AND -bulls AND -puppy AND -pup AND -kitten AND -kitty AND -pussy AND -bovine AND -"eye brow" AND -eyebrow* AND -"eye brows" AND -"eye shadow" AND -mascara AND -makeup AND -eyeliner AND -"eye liner" AND -fart AND -farts AND -farted AND -farting AND -costas AND -"bob costa's" AND -"bob costa" AND -#bobcosta* AND -#costas* AND -#sochi* AND -bobcosta* AND -sochi* AND -olympic* AND -#olympic* AND -sochi AND -olympic AND -brewers AND -@justin* AND -#justin* AND -@florde* AND -justin* AND -bieber AND -belieber AND -@PimpBillClinton AND -@michael5sos AND -michael5sos AND -#michael5sos AND -#vpdebate* AND -#debates2020 AND -#pence AND -#vicepresidentialdebate2020 AND -#vpdebates AND -#pencepinkeye AND -#pencefly AND -#flygate AND -#fly2020 AND -pence AND -debate AND -vpdebate AND -bidenharris2020 AND -debates2020*

Additional tables

Table S1: Selected actual GPT 3.5 and 4 responses to Prompt 2 (YES, NO, MAYBE) for typical Tweets from our dataset (for this table, actual usernames, location and other identifiers were modified to avoid identifying information). NOTE: Prompt 1 elicited probability percentages from Table 2 are provided in square brackets, for comparison to these Prompt 2 results.

Tweet	GPT 3.5 response	GPT 4 response
All undergrads under me got conjunctivitis. So not allowing to theatre and class. Some who r nt effected also act like they got. Managing class all alone. Im jealous seeing them enjoying.	YES conjunctivitis outbreak. The tweet clearly states that multiple undergrads have contracted conjunctivitis and that it is affecting the class and theatre activities. The fact that some who are not affected are pretending to be, suggests that it may be spreading. Conjunctivitis is the type of disease mentioned in the tweet. [Prompt 1 from Table 2 probability value elicited was: 70%]	YES conjunctivitis outbreak. The tweet indicates that multiple undergraduate students have contracted conjunctivitis, and it is affecting their ability to attend class and other activities. The mention of "all undergrads under me" suggests that there is more than one case and it is spreading among the students. [Prompt 1 from Table 2 probability value elicited was: 60%]

Pharmacies ran out of eye drops for conjunctivitis as those infected continue to go to work, school, gatherings, and so on. They affect others–urgh!	YES conjunctivitis outbreak. The tweet mentions that pharmacies ran out of eye drops for conjunctivitis, indicating that there is a high demand for treatment, which suggests that there are more than one case of conjunctivitis. Additionally, the tweet mentions that those infected continue to go to work, school, and gatherings, which implies that the disease is spreading. Therefore, it is likely that there is a conjunctivitis outbreak occurring at the time the tweet was made. [Prompt 1 from Table 2 probability value elicited was: 80%]	YES conjunctivitis outbreak. The tweet indicates that there are multiple cases of conjunctivitis, as pharmacies have run out of eye drops and people continue to go to work, school, and gatherings, potentially spreading the infection. [Prompt 1 from Table 2 probability value elicited was: 80%]
@user conjunctivitis??	MAYBE conjunctivitis outbreak. The tweet only mentions "conjunctivitis" without any further information about the number of cases or spread. It is uncertain whether there is an outbreak or not. [Prompt 1 from Table 2 probability value elicited was: 50%]	MAYBE conjunctivitis outbreak (uncertain, the tweet indicates maybe 2 or more cases of conjunctivitis, maybe spreading) Explanation: The tweet only mentions "conjunctivitis??" which is not enough information to determine if it is about a large multiperson outbreak or not. It is uncertain and could possibly be referring to more than one case, but there is not enough context to be sure. [Prompt 1 from Table 2 probability value elicited was: 10%]
１日家にいたーひさびさのひきこもりやっぱ結膜炎気になって つきっきりしてたー だいぶ寝たー	MAYBE conjunctivitis outbreak. The tweet mentions "結膜炎" which is the Japanese term for conjunctivitis. However, there is no mention of other people being infected or the disease spreading, so it is uncertain if it is a large multiperson outbreak or just a single case. [Prompt 1 from Table 2 probability value elicited was: 10%]	NO, not conjunctivitis outbreak The tweet seems to be talking about a single person staying at home due to concerns about conjunctivitis and spending time resting. There is no indication of a large multiperson outbreak or any evidence of the disease spreading [Prompt 1 from Table 2 probability value elicited was: 10%]
do i have pink eye or could it be my allergies again?	NO, not conjunctivitis outbreak. This tweet is about an individual experiencing symptoms of pink eye or allergies, but there is no indication of a larger outbreak or spread of the disease. [Prompt 1 from Table 2 probability value elicited was: 0%]	NO, not conjunctivitis outbreak. The tweet only mentions a single individual questioning whether they have conjunctivitis or allergies, with no indication of other cases or spreading. [Prompt 1 from Table 2 probability value elicited was: 5%]
Did you know that the first case of viral conjunctivitis was found in 1902? It is 2020 and "science" still has no "cure" for it!	NO, not conjunctivitis outbreak. The tweet is not indicating any current outbreak or spread of conjunctivitis. It is simply providing historical information about the first case of viral conjunctivitis. [Prompt 1 from Table 2 probability value elicited was: 0%]	NO, not conjunctivitis outbreak (the tweet is irrelevant or indicates 0-1 cases of conjunctivitis max, not spreading or not occurring at the time the tweet was made) Explanation: The tweet is discussing the history of viral conjunctivitis and the lack of a cure, but it does not mention any current outbreak or cases of the disease. [Prompt 1 from Table 2 probability value elicited was: 0%]

Table S2: Correlation of weekly Tweet volume and elicited outbreak percentages by the LLMs excluding weeks with zero Tweets meeting our search criteria. 95% confidence intervals shown in parentheses.

**Country**	**GPT 3.5 weekly mean, r (95% CI)**	**GPT 4 weekly mean, r (95% CI)**	**GPT 3.5 YES, *r*, (95% CI)**
India	0.02 (-0.08, 0.28)	0.09 (0.01, 0.38)	0.01 (-0.03, 0.16)
Guam	0.48 (0.40, 0.64)	0.67 (0.55, 0.83)	0.05 (0.02, 0.10)
Martinique	0.47 (0.39, 0.74)	0.56 (0.51, 0.85)	0.20 (0.12, 0.73)
Philippines	0.21 (0.11, 0.32)	0.28 (0.18, 0.37)	0.04 (-0.05, 0.18)
American Samoa	0.52 (0.35, 1.00)	0.70 (0.58, 1.00)	0.17 (0.08, 1.00)
Fiji	0.30 (0.27, 0.72)	0.36 (0.30, 0.81)	0.14 (0.12, 0.97)
Costa Rica	0.15 (0.12, 0.38)	0.20 (0.14, 0.51)	0.07 (0.02, 0.17)
Haiti	0.13 (0.09, 0.41)	0.26 (0.22, 0.69)	0.05 (0.02, 0.13)
Bahamas	0.39 (0.33, 0.52)	0.56 (0.51, 0.70)	0.08 (0.04, 0.17)
